# T Cell Responses to Mycobacterial Glycolipids: On the Spectrum of “Innateness”

**DOI:** 10.3389/fimmu.2020.00170

**Published:** 2020-02-11

**Authors:** Charlotte A. James, Chetan Seshadri

**Affiliations:** ^1^Molecular Medicine and Mechanisms of Disease (M3D) PhD Program, Department of Pathology, School of Medicine, University of Washington, Seattle, WA, United States; ^2^Department of Medicine, School of Medicine, University of Washington, Seattle, WA, United States; ^3^Tuberculosis Research and Training Center, School of Medicine, University of Washington, Seattle, WA, United States

**Keywords:** mycobacteria, glycolipids, lipid antigen, T cell, CD1, tuberculosis, diagnostics, vaccines

## Abstract

Diseases due to mycobacteria, including tuberculosis, leprosy, and Buruli ulcer, rank among the top causes of death and disability worldwide. Animal studies have revealed the importance of T cells in controlling these infections. However, the specific antigens recognized by T cells that confer protective immunity and their associated functions remain to be definitively established. T cells that respond to mycobacterial peptide antigens exhibit classical features of adaptive immunity and have been well-studied in humans and animal models. Recently, innate-like T cells that recognize lipid and metabolite antigens have also been implicated. Specifically, T cells that recognize mycobacterial glycolipid antigens (mycolipids) have been shown to confer protection to tuberculosis in animal models and share some biological characteristics with adaptive and innate-like T cells. Here, we review the existing data suggesting that mycolipid-specific T cells exist on a spectrum of “innateness,” which will influence how they can be leveraged to develop new diagnostics and vaccines for mycobacterial diseases.

## Introduction

Mycobacterial diseases such as tuberculosis (TB), leprosy, and Buruli ulcer, are of high global health importance and disproportionately affect individuals in resource-limited settings. A major challenge to reducing the global burden of these diseases is the lack of effective diagnostics and vaccines. Several studies in murine and non-human primate models implicate T cells as necessary to control *Mycobacterium tuberculosis* (M.tb) (1, 2). Specifically, these studies led to a focus on T cells that recognize peptide antigens presented by major histocompatibility complex (MHC) molecules, in part because MHC class II (MHC-II) knockout mice infected with M.tb had a significantly shorter life-span than MHC-I knockout or wild-type mice (2). Consequently, the majority of new vaccine development efforts have been focused on eliciting robust CD4 T cell responses to mycobacterial protein antigens (3, 4).

**Figure 1:**
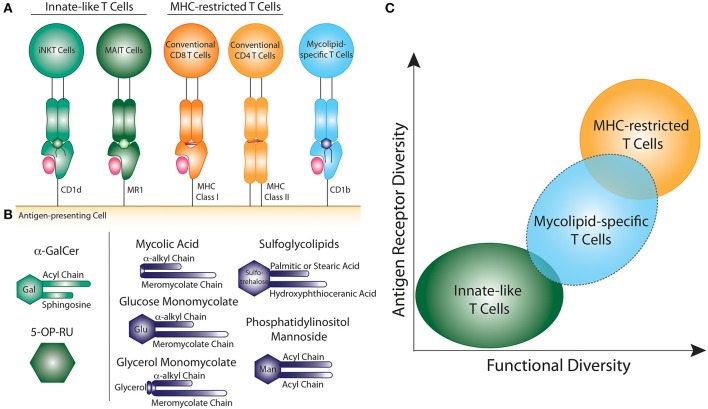
Comparison of innate-like, MHC-restricted, and mycolipid-specific T cells. **(A)** Graphical depiction of innate-like (green), MHC-restricted (orange), and mycolipid-specific T cells (blue). **(B)** Graphical depictions of canonical innate-like antigens or mycolipid antigens presented by CD1b. α-galactosyl ceramide (α-GalCer) is recognized by iNKT cells when presented by CD1d and is comprised of a galactose head group and a sphingosine and acyl chain. 5-(2-oxopropylideneamino)-6-D-ribitylaminouracil (5-OP-RU) is recognized by MAIT cells when presented by MR1. Mycolic acid is comprised of an α-alkyl and meromycolate chain. Glucose monomycolate and glycerol monomycolate are comprised of a mycolic acid base with a glucose and glycerol head group, respectively. Sulfoglycolipids are comprised of a sulfated trehalose headgroup and an hydroxyphthioceranic acid and palmitic acid or stearic acid chains. Phosphatidyl-*myo*-inositol mannoside is comprised of two acyl chains and two or more mannose residues as a headgroup. **(C)** Summary of the key determinants of T cell “innateness”. Innate-like T cells (green) have low antigen receptor diversity and fewer functional subsets. Peptide-specific T cells (orange) exhibit high antigen receptor diversity and higher functional diversity and plasticity. Mycolipid-specific T cells (blue) have qualities that overlap with both innate-like and MHC-restricted T cells, but are functionally more similar to MHC-restricted T cells.

However, T cells can also recognize non-peptide antigens, such as lipids and microbial metabolites, presented by cluster of differentiation 1 (CD1) and MHC-related protein 1 (MR1), respectively (5, 6) (Figure 1A). These CD1- and MR1-restricted T cells have been largely defined as “innate-like” T cells due to their unique biology, as we describe below. The two major populations of CD1- and MR1-restricted T cells are invariant natural killer T (iNKT) cells and mucosal associated invariant T (MAIT) cells, respectively. Notably, iNKT and MAIT cells represent only a subset of T cells that recognize non-peptide antigens. Here, we focus our comments on the potential role of T cells that respond to mycobacterial cell surface lipid (mycolipid) antigens. We describe their origins, phenotypes, and functions, with particular attention to data emerging from human studies. We also provide context for the existing data with respect to a large body of literature examining T cells with adaptive or innate-like phenotypes. Our analysis suggests that mycolipid-specific T cells lie somewhere in the middle of this spectrum, which will influence how we leverage their unique biology to improve the diagnosis and treatment of mycobacterial diseases.

### Mycolipids as T Cell Antigens

Lipids found in the mycobacterial cell wall, collectively termed mycolipids here, have been shown to bind CD1 molecules and activate human T cells (5, 7, 8) (Figure 1B). Long-chain mycolipids preferentially bind to CD1b due to its large binding pocket, while comparatively shorter lipids, and lipopeptides are presented by CD1c and CD1a, respectively (9–11). Mycolic acid (MA) was the first mycobacterial lipid antigen shown to be recognized by T cells via the CD1b antigen presentation pathway (5). Since then, glucose monomycolate (GMM), and glycerol monomycolate (GroMM) were discovered as glycated forms of mycolic acid that are also recognized as T cell antigens (12, 13). In addition to the mycolates, lipoarabinomannan (LAM), and its constituent lipid phosphotidyl-*myo*-inositol mannoside (PIM), are abundant cell surface mycolipids recognized by human T cells (14, 15). Finally, diacylated sulfogylcolipids (SGLs), which are uniquely expressed in the cell wall of virulent strains of M.tb, are also T cell antigens (16). Thus, six structurally defined mycolipids are presented by CD1b to human T cells (Figure 1B). In addition, mannosyl-β-1-phosphoketide (MPM) is restricted by CD1c (17) and didehydroxymycobactin (DDM) is a lipopeptide antigen presented by CD1a (10).

T cell responses to many of these antigens have been detected among individuals infected with M.tb and *Mycobacterium leprae*, the causative agents of TB and leprosy, respectively. *In vitro*-derived T cell lines have been isolated from peripheral blood and lesion biopsies of individuals infected with *M. leprae* (15, 18). GMM- and SGL-specific T cells have been detected at a higher frequency in individuals infected with M.tb than healthy individuals (16, 19). Mycolipid-specific T cells have also been studied directly from the peripheral blood of individuals with active TB (20).

### Mycolipid-Specific T Cell Receptor Diversity

As a result of MHC polymorphism, MHC-restricted T cells exhibit diverse TCRs that are unlikely to be shared across individuals, even for a specific peptide antigen. By contrast, the CD1 and MR1 antigen presentation systems are highly conserved across human populations (21, 22). Thus, one defining feature of iNKT and MAIT cells is the expression of a semi-invariant TCR that is shared across genetically unrelated individuals. The canonical TCR that is expressed by iNKT cells includes an invariant TCR-α chain that is comprised of a TRAV10/TRAJ18 gene rearrangement (23, 24). The canonical TCR expressed by MAIT cells expresses a TCR-α chain comprised of a TRAV1-2/TRAJ33,12,22 gene rearrangements (24–26). While MHC-restricted T cells are typically activated by a single peptide antigen, iNKT and MAIT cells that express the canonical invariant TCR-α chain have the ability to recognize multiple ligands. iNKT cells are defined by their recognition of α-galactosylceramide (α-GalCer), which is a potent and high affinity antigen (27, 28). However, iNKT cells can also recognize a variety of other antigens presented by CD1d, such as lyso-phospholipids, glycosphingolipids, and sulfatides (29–32). The canonical ligand for MAIT cells is 5-(2-oxopropylideneamino)-6-D-ribitylaminouracil (5-OP-RU) presented by MR1, which is recognized with high affinity (6, 33). MAIT cells can also recognize chemically diverse small molecules such as drugs, drug metabolites, and drug-like molecules (34).

The development of CD1b tetramers for GMM, SGLs, and MA have enabled the isolation of mycolipid-specific T cells to study the T cell receptor (TCR) repertoire and functional diversity directly *ex vivo* (35–40). TCR diversity among T cells specific for mycolipids has been best studied using GMM as the model antigen. Like iNKT and MAIT cells, a subset of GMM-specific T cells present in genetically unrelated donors expresses a semi-invariant TCR. This TCR is termed the germline-encoded mycolyl-reactive (GEM) TCR and includes a conserved TCR-α that consists of a TRAV1-2/TRAJ9 gene rearrangement (38). However, recent data suggest that GMM-specific T cells expressing this conserved TCR may actually constitute the minority of GMM-specific T cells, which have been shown to express a more diverse TCR repertoire (35, 39). GMM-specific TCRs have also been shown to display varying levels of affinity and specificity for GMM. A GMM-specific T cell clone with a TCR that uses TRAV1-2 has a higher affinity for GMM-CD1b tetramer and expresses a similar but distinct cytokine profile than a T cell clone that does not use TRAV1-2 (39). Further, the GMM-specific T cell clone LDN5 displays such fine specificity that alterations to the sugar head group or lipid tail moieties distal to the head group can abrogate antigenicity (13). A different GMM-specific T cell clone expressing the semi-invariant TCR-α chain was shown to recognize both GMM and MA, which lacks a sugar head group (38, 41). The mechanism for this promiscuity is not fully understood but may relate to modulation of the CD1b surface by the embedded lipid tails (42). Future studies comparing TCR sequences may reveal whether a molecular or structural pattern is responsible for the TCR being permissive to multiple mycolate ligands.

Of note, NKT cells can also express diverse TCRs and are collectively referred to as Type II iNKT cells (43, 44). Similarly, T cells that are restricted by MR1 can also express TCRs that do not contain the TRAV1-2 gene segment (45, 46). This is similar to the pattern of GMM-specific TCR diversity that has been described above as some GMM-specific T cells express a semi-invariant TCR, but others exhibit diverse TCRs.

Thus, GMM-specific TCRs exhibit properties of both innate and adaptive T cells. On the one hand, they are characterized by diverse TCRs with varying affinity, but there is also a dominant and conserved semi-invariant TCR that is shared across individuals. While several mycolipid-restricted TCRs have been identified, further analysis of large groups of T cells for each lipid antigen is required to determine if common TCR genes or motifs are shared between mycolipid-specific T cells from unrelated donors. This can be accomplished by single cell RNA-sequencing or targeted TCR sequencing of mycolipid-specific T cells.

### Mycolipid-Specific T Cell Development

MHC-restricted T cells undergo positive selection in the thymus by thymic epithelial cells and egress as naive T cells (47, 48). By contrast, iNKT cells and MAIT cells undergo positive selection by other thymocytes that express CD1d or MR1 on their cell surface, respectively (49, 50). iNKT cells acquire an innate-like effector memory program during thymic development and egress from the thymus expressing the transcription factor promyelocytic leukemia zinc finger (PLZF), which regulates an innate-like phenotype in iNKT and MAIT cells, as well as innate lymphoid and natural killer cells (51–53). MAIT cells egress from the thymus with a naive surface phenotype and acquire an innate-like effector memory program, including PLZF expression, during expansion in the periphery following microbial colonization (52, 54). The development of mycolipid-specific T cells is largely unknown. In a human transgenic mouse model, MA-specific T cells were selected by thymocytes that express CD1b rather than thymic epithelial cells, similar to iNKT and MAIT cells (55). However, it has been shown that murine mycolic acid-specific T cells do not express PLZF (55). Whether human mycolipid-specific T cells express PLZF is unknown.

### Mycolipid-Specific T Cell Activation and Memory Phenotype

Naive MHC-restricted T cells are present at low frequency and require TCR ligation by their cognate antigen as well as additional stimulation by professional antigen presenting cells to become activated (56–58). These T cells will then undergo a rapid expansion, up to 25-fold in 72 h, and differentiate into effector T cells (59). Upon clearance of antigen, the effector T cell population will then contract and form long-lived memory T cells that have enhanced proliferative potential and more rapid activation and cytokine production upon subsequent encounter with antigen (60).

By contrast, iNKT and MAIT cells do not necessarily require cognate recognition of antigen to be activated in the periphery, and they acquire their effector memory phenotype during development, as described above. For example, bacterial infection and toll-like receptor (TLR) agonists can induce interferon gamma (IFN-γ) production in MAIT cells, through an interleukin (IL-)12- and IL-18-dependent mechanism (61). It was also shown that MAIT cells derived from human peripheral blood produce IFN-γ and tumor necrosis factor (TNF) when stimulated with IL-12, IL-15, and IL-18 (62). Further, TCR signaling is not sufficient for MAIT cell activation in the absense of these inflammatory signals (62). iNKT cells express high levels of IL-12 receptor, and IFN-γ production during *in vitro* stimulation is dependent on IL-12 signaling (63). In the absence of activation, iNKT and MAIT cells are present at high frequency, comprising up to 1 and 10% of circulating T cells, respectively (64–66). However, they have an impaired proliferative potential compared to MHC-restricted T cells after stimulation (67).

It is unknown what factors are required for activation of naive mycolipid-specific T cells. Mycolipid-specific T cells are found at a higher frequency in individuals with documented exposure to mycobacteria, suggesting that prior antigen exposure drives activation and proliferation (16, 19). There is evidence that immunization of cattle with GMM produces GMM-specific T cells with enhanced proliferative potential, also suggesting that exposure to antigen can lead to activation and enhanced recall responses (68). In addition, a study of GMM-specific T cells directly *ex vivo* showed that over 80% of this population expresses CD45RO, further suggesting that they are antigen experienced (69). The proportion of different memory subsets found in antigen-experienced individuals is currently unknown, and whether antigen experience is required for memory phenotype acquisition also remains to be determined (Table 1). This could be comprehensively examined by looking at the memory phenotype of mycolipid-specific T cells using CD1 tetramers in longitudinal cohort studies and in animal models pre- and post-antigen exposure. Other metrics of T cell memory should also be investigated, such as enhanced proliferative potential, clonal expansion, and enhanced secondary recall responses. Finally, it is unknown whether mycolipid-specific T cells can be activated in a cytokine-driven and TCR-independent manner (Table 1). This could be investigated using *in vitro* activation studies such as IFN-γ secretion or activation marker upregulation (e.g., CD69) as assessed by flow cytometry.

**Table 1:** Summary of properties exhibited by innate-like, MHC-restricted, and mycolipid-specific T cells.

**Topic**	**Innate-like** **T cells**	**MHC-restricted** **T cells**	**Mycolipid-specific** **T cells**
TCR diversity	Semi-invariant	Highly diverse	Semi-invariant and highly diverse
TCR promiscuity	Yes	No	Yes
Activation requirements	TCR dependent and independent	TCR dependent	TCR dependent?
Memory phenotype	Acquired during development	Acquired after development	Unknown
Thymic selection	Dependent on thymocytes	Dependent on thymic epithelium	Dependent on thymocytes
Functional profiles	Th1, Th17	CD4 (Th1, Th2, Th17) CD8 (CTL)	Th1 and CTL, some Th17

*This table compares the major aspects of T cell biology between innate-like T cells, MHC-restricted T cells, and mycolipid-specific T cells. TCR diversity is evaluated by conservation of TCR sequences across genetically unrelated donors. TCR promiscuity is defined as the ability of the same TCR to recognize several chemically unrelated ligands. The activation requirements for T cell subsets are described as the reliance on TCR ligation for T cell activation. As all three T cell subsets have been shown to exhibit T cell memory in some capacity, we summarized the differences between memory phenotype as acquisition during or after T cell development. Thymic selection describes the cells that are involved in the selection of these T cell subsets during development. Lastly, the functional profiles for these subsets are summarized as the major functional profiles that have been described for T cells in each subset*.

### Functional Profiles of Mycolipid-Specific T Cells

The phenotypes of MHC-restricted T cells are broadly dictated by the inflammatory environment, TCR co-receptor expression, and in some cases, TCR affinity (70). Largely, these functional categories are pro-inflammatory (Th1, Th2, and Th17), cytotoxic, or regulatory (Treg). The pro-inflammatory subsets are regulated by the transcription factors T-bet, GATA3, and ROR-γt, respectively (71–73). Regulatory T cells are regulated by the transcription factor FoxP3 (74, 75). However, there is evidence that MHC-restricted T cells exhibit functional plasticity and can alter their functional program over the course of the T cell's life-span (76).

iNKT cells are largely characterized by constitutive IFN-γ expression and IL-4 production, a phenotype not commonly exhibited by MHC-restricted T cells (77, 78). MAIT cells are capable of secreting a variety of cytokines, such as IL-17, IFN-γ, IL-2, and TNF (64). The functional subsets of iNKT and MAIT cells are largely defined by the magnitude of PLZF expression and the expression of the master transcription factors that define MHC-restricted T cell functional subsets (77, 79, 80). However, no Th2 equivalent has been identified for MAIT cells (80, 81).

Most of the data regarding mycolipid-specific T cells have been obtained through studies of *in vitro*-derived T cell clones, which have been shown to express IFN-γ, TNF, and IL-17, as well as lyse M.tb-infected cells and control M.tb replication *in vitro* (13, 16, 18, 38, 55, 82). T cells with different affinities for GMM-loaded CD1b tetramers have been shown to have different functional capabilities. The high affinity GMM-specific T cells expressed high levels of Th1 cytokines, whereas the lower affinity T cells expressed Th1 cytokines along with IL-17 (39). The most comprehensive study performed on cells stimulated directly *ex vivo* showed that CD4^+^ mycolipid-specific T cells secrete IFN-γ, TNF, IL-2, and IL-17 in polyfunctional combinations (83). Notably, different lipid antigens yielded unique functional profiles with GMM-specific T cells being the most polyfunctional, while glycerol monomycolate-specific T cells largely expressed IL-2 alone (83). Whether CD4^+^ mycolipid-specific T cells can be categorized into traditional T-helper lineages based on expression of canonical transcription factors is currently unknown, as are the factors that govern acquisition of specific functions. These questions can be investigated in human cohorts using tetramers and intranuclear flow cytometry or RNA sequencing as well as animal studies to determine how these functional profiles are acquired. In addition, the diversity of functions exhibited by mycolipid-specific T cells could be comprehensively examined using single-cell RNA sequencing.

iNKT cells and MAIT cells may contribute to protection from M.tb based on data derived from *in vitro* and *in vivo* models of TB. Patients with active TB have reduced levels of iNKT and MAIT cells in peripheral blood or bronchoalveolar lavage samples when compared to healthy donors and individuals with latent TB, suggesting that these cells home to the lung to mediate a protective immune response (84–87). In a murine model, pulmonary mycobacterial infection leads to recruitment of MAIT cells in the lung, and this was associated with reduced bacterial burden during infection (88). While MAIT cells from this study exhibited diverse functional profiles, no particular functional profile was associated with reduction in disease burden (88). iNKT cells can recognize infected macrophages and kill intracellular mycobacteria in a granulocyte-macrophage colony-stimulating factor (GM-CSF) dependent manner (89, 90). The role of mycolipid-specific T cells in mediating protection has been evaluated in two animal models. Guinea pigs immunized with purified or synthetic mycobacterial lipids showed reduced lung pathology upon subsequent challenge with M.tb (91, 92). In a humanized CD1 transgenic mouse model, MA-specific T cells were adoptively transferred, trafficked to the lung during M.tb challenge, and led to decreased bacterial burden in a CD1-dependent manner (55). Interestingly, MA-specific T cells were activated earlier in the course of M.tb infection than MHC class II-restricted T cells that recognized antigen 85B (Ag85B) (55). MA-specific T cells isolated from the lung secreted TNF, IFN-γ, IL-2, and expressed CD107a, a surrogate marker of cytotoxic activity (55). Taken together, these data suggest that mycolipid-specific T cells can provide protection from mycobacterial infection, given they efficiently traffic to the lung during the early stages of infection. However, future studies should investigate which among the various lipid antigens are recognized by protective T cells, what functional profiles these T cells exhibit, and how to elicit these T cell responses through vaccination in humans and animal models.

In summary, several factors suggest that mycolipid-specific T cells may be more like MHC-restricted T cells than iNKT or MAIT cells in their functional qualities (Figure 1C). These include a low precursor frequency, increased frequency in mycobacteria-infected individuals, lack of PLZF expression, and the preliminary evidence of antigen-dependent activation, proliferation, and memory phenotype acquisition. However, some studies have shown that a subset of mycolipid-specific T cells exhibit innate-like qualities as well, such as expression of a semi-invariant TCR and promiscuous ligand recognition. On the whole, mycolipid-specific T cells have qualities of both innate-like and MHC-restricted T cells and exist on a spectrum of “innateness” (Figure 1C). Future studies should be directed at understanding their development in humans and animal models, *ex vivo* functional studies in humans, and how memory phenotypes are modulated by antigen exposure (Table 1).

## Discussion

The diagnosis of mycobacterial disease could be improved by leveraging the biology of mycolipid-specific T cells. Latent TB is typically diagnosed by an interferon gamma release assay, which measures T cell responses to secreted protein antigens but is a poor predictor for progression to active disease (93). The diagnosis of leprosy and Buruli ulcer are established by identifying bacilli in affected skin lesions, but this often results in false negative results because of the paucibacillary nature of these diseases. Notably, diagnostics targeting cell surface mycolipids are already being used for both TB and leprosy. High-affinity IgG antibodies targeting LAM have been incorporated into a urine-based assay for TB and have shown clinical utility in the setting of HIV co-infection (94). IgM antibodies targeting phenolic glycolipids (PGLs) are being used for the diagnosis of leprosy (95). Even though the current standard for leprosy diagnosis incorporates IgM antibodies to PGL, this could improve as PGLs also likely contain T cell epitopes, which could be leveraged to develop higher affinity IgG antibodies to PGLs. This is also probably true of LAM, currently the target of antibody-based diagnostics for TB, which actually contains a T cell epitope in the form of PIM (14). Finally, diagnostics could be improved by targeting specific lipids, such as SGLs, which have been shown to be preferentially expressed by M.tb and are absent from BCG and environmental strains (96, 97).

One approach to further improve diagnostics would be to advance our understanding of how mycolipid-specific T cells influence the generation of these mycolipid-specific antibodies. Notably, CD4 GMM- and MA-specific T cells express CD40 ligand (CD40L), which suggests that these T cells may be involved in B cell maturation and immunoglobulin class-switching (83). iNKT cells have previously been shown to provide both cognate and non-cognate help to B cell class switching and affinity maturation (98–100). Similarly, supernatants from activated MAIT cells promote B cell differentiation into plasmablasts, and production of IgA, IgM, and IgG antibodies (101). It is possible that mycolipid-specific T cells provide both cognate and non-cognate help to B cells.

Though we have focused our comments on mycolipids as T cell antigens, it is important to note that these lipids may also have adjuvant properties. Indeed, many of these antigens are structurally similar to adjuvants in clinical use, such as GLA-SE and trehalose dibehenate (TDB) (102, 103). Some T cell antigens, such as phosphotidyl-*myo*-inositol mannosides, may fortuitously act as both antigen and adjuvant by activating Toll-like receptor pathways (14, 104).

An emerging approach to the diagnosis of infectious diseases is using high-throughput sequencing of TCRs that are expanded in peripheral blood. Using machine learning, a recent study showed that a TCR “fingerprint” of cytomegalovirus (CMV) infection could be identified (105). This is a notable result because most CMV-specific TCRs would be private due to the highly polymorphic nature of MHC. On the other hand, CD1-restricted TCRs are expected to be public due to the non-polymorphic nature of CD1. Indeed, our group found that both shared and private TCR repertoires of GMM-specific T cells were associated with active TB disease in a South African cohort (35). Thus, TCRs specific for mycolipids could be developed into a blood-based diagnostic tool.

Mycolipid-specific T cells also hold significant promise for new vaccine approaches. There is no licensed vaccine for either leprosy or Buruli ulcer. The only licensed vaccine for TB is *Mycobacterium bovis* bacille Calmette-Guérin (BCG). This vaccine protects children from disseminated forms of TB but shows variable efficacy in preventing pulmonary TB in adults (106–108). As individuals with pulmonary TB are infectious, the epidemic cannot be controlled with BCG, and new TB vaccines are urgently needed. Immunizing with mycolipids has conferred modest protection to TB in rodent models (91, 92). The next step to increasing vaccine efficacy should be to ensure inclusion of a variety of antigenic types. One strategy of increasing antigenic breadth is by utilizing live-attenuated or killed whole cell vaccines (109). If T cell responses to mycolipid antigens are associated with positive outcomes in these studies, then using lipid-subunit vaccines to target a specific population can also be considered as a novel vaccine strategy. Natural candidates for lipid-subunit vaccine are SGLs, which have only been detected in virulent strains of M.tb (97). Further, SGL-specific T cells are expanded in individuals who have been exposed to M.tb (16).

Thus, mycolipid-specific T cells appear to bridge innate-like and adaptive T cell biology and should be considered for novel diagnostic and vaccination strategies targeting mycobacterial diseases.

## Author Contributions

CJ and CS wrote and edited the manuscript.

### Conflict of Interest

The authors declare that the research was conducted in the absence of any commercial or financial relationships that could be construed as a potential conflict of interest.
